# Greening the future of healthcare: implementation of sustainability strategies in German hospitals and beyond—a review

**DOI:** 10.3389/fpubh.2025.1559132

**Published:** 2025-05-02

**Authors:** Roxana Schwab, Lina Judit Schiestl, Annette Hasenburg

**Affiliations:** Department of Obstetrics and Gynecology, University Medical Center of the Johannes Gutenberg University Mainz, Mainz, Germany

**Keywords:** sustainability, healthcare, well-being, energy, climate protection, greenhouse gas emissions, planetary health, planetary health diet

## Abstract

This study explores the implementation of sustainability measures within German hospitals, emphasizing their critical role in mitigating environmental impacts and promoting public health. The healthcare sector significantly contributes to global greenhouse gas emissions, necessitating urgent reforms in energy use, waste management, construction, transportation, and food systems. Key findings highlight the potential for hospitals to enhance energy efficiency, adopt renewable energy sources, and reduce waste through innovative technologies and sustainable practices. Initiatives such as green hospital designs, climate-conscious food systems, and sustainable procurement strategies are central to reducing the ecological footprint. Despite these opportunities, barriers such as workforce shortages, insufficient funding, and technical complexity hinder progress. Addressing these challenges through leadership commitment, resource allocation, and staff engagement is essential for aligning the healthcare sector with national and international sustainability goals. By prioritizing sustainability, hospitals can achieve long-term economic benefits, improve patient outcomes, and foster a healthier, more resilient society.

## Highlights

- Comprehensive analysis of sustainability initiatives in German hospitals, structured around five key sustainability objectives.
- Identifies major barriers (workforce shortages, financial constraints, and regulatory complexity) hindering sustainability adoption.
- Compares Germany’s approach to hospital sustainability with international case studies (UK, USA, and Australia).
- Highlights cost-effective strategies for reducing hospital carbon footprints, including energy-efficient infrastructure, waste reduction, and sustainable procurement.
- Provides policy recommendations for policymakers, hospital administrators, and healthcare professionals to accelerate sustainability initiatives.

## 1 Introduction

Environmental protection and sustainability are among the primary responsibilities of every physician. The German Professional Code for Physicians states, “The task of physicians is to preserve life, protect and restore health, alleviate suffering, assist the dying, and contribute to the preservation of the natural fundaments of life with regard to their significance for human health” (1).

We are currently at a crossroads regarding the distribution of naturally occurring resources. On one hand, the global population is increasing, simultaneously asserting legitimate claims for a fair distribution of available resources. On the other hand, natural resources such as non-renewable minerals and fossil raw materials, clean water, and a clean atmosphere are limited. Consequently, enforcing local, national, and international sustainability goals is becoming a primary target of environmental policy, economics, and social sciences on various levels.In response to these challenges, the United Nations (UN) adopted the 2030 Agenda for Sustainable Development in 2015, which outlines 17 Sustainable Development Goals (SDGs) aimed at addressing global issues such as poverty, inequality, environmental degradation, and climate change. These goals provide a universal framework for achieving social, economic, and environmental sustainability by 2030 (2). In 2016, the Paris Agreement on climate protection came into effect, committing signatory countries, including Germany, to limit global warming to a maximum of 2°C, but preferably below 1.5°C, relative to the pre-industrial era (3). As part of the European Union’s Green Deal Agenda, Europe has committed to becoming the first climate-neutral continent by 2050. This involves reducing emissions of environmentally harmful substances by 55% by 2030 compared to 1990 (4). Furthermore, the capacity of renewable energies is set to increase by at least 42.5%, and energy efficiency is targeted to improve by at least 11.7% until 2030 (4).

The German federal government has presented its sustainability strategy in alignment with international and European resolutions (5). In 2021, the German federal government established six paramount goals: well-being, social justice, energy transition and climate protection, sustainable construction and transportation, sustainable agricultural and food systems, and a pollution-free environment (6).

The German healthcare sector is progressively gaining significance within the national economy. As of 2022, the healthcare sector accounted for 12.7% of the total gross value added and engaged 17.7% of the workforce in Germany (7). Almost 10% of Germany’s total exports can be traced back to the healthcare sector (7). Moreover, the healthcare sector experienced an annual growth rate of approximately 3.9% in recent years (7). In 2022, the gross value added within the German hospital sector reached 78.2 billion euros (8).

Sustainability has become a key factor in hospital management, influencing not only environmental outcomes but also economic efficiency and competitiveness. As demonstrated in a current study, hospitals increasingly focus on energy-efficient infrastructure, resource optimization, and digitalization to reduce costs while meeting sustainability targets. However, while German hospitals score high in social quality metrics, environmental and organizational sustainability require further improvement (9).

**Figure 1:**
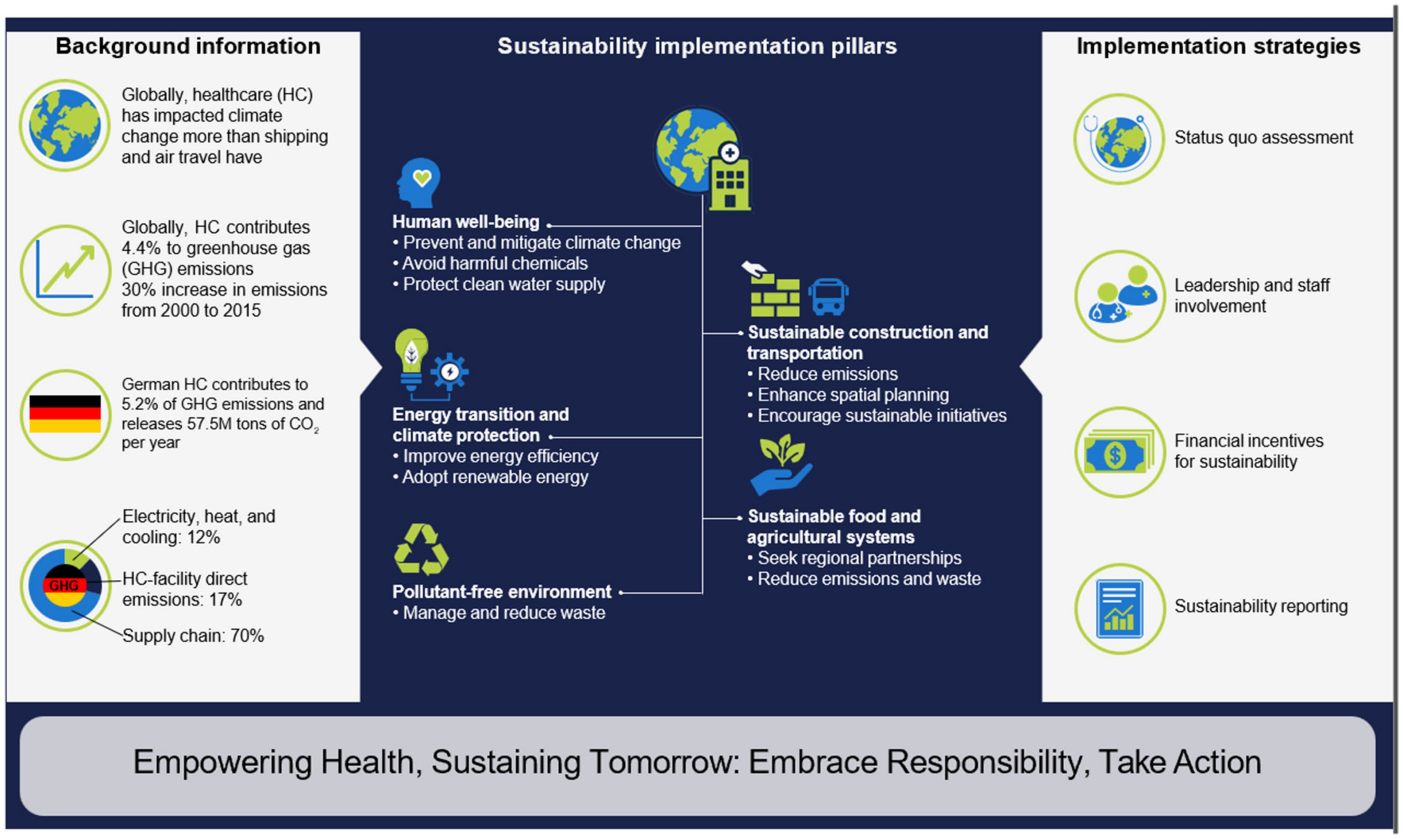
Towards greener health: building a sustainable future for German healthcare. HC: healthcare; GHG: greenhouse gas emissions.

The global healthcare sector contributes significantly to net greenhouse gas emissions, accounting for 4.4% of the total, with major contributions from the USA, China, and the European Union (Figure 1) (10, 11). In Germany, this contribution is 5.2%, corresponding to an annual emission of 57.5 million tons of medical CO_2_ (11, 12). The USA reports an even higher proportion at 9.8% (11). A recent comprehensive review disclosed a nearly 30% global increase in greenhouse gas emissions from the healthcare sector between 2000 and 2015 (Figure 1) (13). CO_2_ emissions dominate among greenhouse gases, constituting 51% (originating from fuel combustion and the fuel used in the supply chains of medical products). Supply chains in healthcare, including the production, transportation, and waste disposal of pharmaceutical products, chemicals, and food, contribute to more than 70% of emissions in the healthcare sector (Figure 1) (11). In 2021, the German government enacted the “Supply Chain Due Diligence Act,” imposing responsibility on companies for all stages, both nationally and internationally, from production to delivery to the end customer, encompassing both environmental and social considerations (14). In 2022, the European Union introduced a draft directive for an EU Supply Chain Law (15).

An additional 17% of emissions from the healthcare sector arise from direct emissions within healthcare facilities, and 12% result from electricity, heat, and cooling (Figure 1) (10). The healthcare sector has a more significant impact on climate change than shipping or air travel (12). However, this relationship is reciprocal, as climate change also causes the emergence and aggravation of existing diseases. For example, premature births or other adverse events in pregnancy occur more frequently due to the increased occurrence of extreme weather conditions (16). Air pollution, industrial chemicals in the food chain, and wastewater can lead to changing hormonal levels, which may lead to fertility issues or even the development of cancer (17). Climate-conscious initiatives in the healthcare/hospital sector are pivotal due to their extensive interactions with other economic sectors and their influence on the current and future social andpolitical well-being, and health of the population. These initiatives can play a crucial role in advancing sustainable healthcare, mitigating greenhouse gas emissions, conserving the environment, and safeguarding health (11). Concurrently, efforts should be made to enhance the importance of primary prevention as a sustainability measurement.

National and international sustainability goals suggest that, by 2030, healthcare services in Germany and worldwide should adopt environmentally friendly practices to safeguard the environment and human health.

Despite increasing efforts to integrate sustainability in healthcare, existing research often lacks a comprehensive analysis of the practical implementation of sustainability measures within hospitals, particularly in Germany. Prior studies primarily focus on isolated aspects, such as energy efficiency, waste management, or the role of healthcare professionals in promoting climate-conscious initiatives. However, a holistic assessment that examines multiple sustainability dimensions—human well-being, energy transition, sustainable infrastructure, food systems, and pollution control—remains underexplored. Additionally, while policy guidelines exist at national and international levels, there is limited analysis of real-world barriers and facilitators that hospitals face in aligning with sustainability goals.

This study addresses these gaps by offering:

- A comprehensive review of sustainability initiatives in German hospitals, structured around five key sustainability objectives outlined by the federal government.
- A critical assessment of both enabling factors and barriers to the adoption of sustainability measures, including workforce shortages, financial constraints, and technical complexities.
- An evaluation of economic feasibility, highlighting cost-saving opportunities and long-term financial benefits for hospitals implementing sustainability measures.
- A policy-oriented perspective, offering recommendations to policymakers, hospital administrators, and healthcare professionals to accelerate sustainability integration in hospital settings.

By synthesizing these elements, this paper provides a unique and structured framework for understanding and advancing sustainability in German hospitals, offering insights applicable to healthcare institutions worldwide.

## 2 Methods

This review employed an integrative, narrative, and targeted literature search strategy rather than a formal systematic review methodology. The aim was to provide a comprehensive, up-to-date overview of sustainability measures in German hospitals, focusing on relevance and recency over exhaustive coverage. The flexibility of the integrative review is especially valuable in interdisciplinary fields like healthcare sustainability, where the research spans diverse areas such as public health, engineering, and policy (18, 19). Narrative reviews are highly effective for synthesizing research from multiple perspectives and generating new insights, as opposed to narrowing the focus on specific predefined research questions in systematic reviews (18, 20).

Searches were conducted in PubMed, Google and Google Scholar, using predefined keywords in English and German language, related to sustainability, healthcare, environmental health, and hospital practices.

Keywords were extracted from the thematic focus areas outlined in the research and include MeSH terms and natural language terms relevant to sustainability and healthcare. The final list included but was not limited to:

- Environmental and Health Topics: Environmental Exposure, Air Pollution, Climate Change, Global Health, Environmental Pollutants, Carbon Footprint, Planetary Health, Human well-being
- Healthcare Sector Terms: Health Care Sector, Hospital, Humans, German Hospitals, Food Service, Hospital, Transportation, Sustainable Building, Waste Management
- Sustainability Concepts: Sustainability, Sustainable Development, Energy Efficiency, Energy Management, Energy Transition, Planetary Health Diet, Legal Regulations

Boolean operators were used to combine terms in both English and German, and filters were applied to prioritize literature published within the last 5 years (2019–2024).

The papers were clustered based on recurring sustainability themes, such as energy efficiency, waste management, and sustainable food systems, with a particular focus on policy recommendations and barriers to implementation. The clustering process was guided by an iterative approach, refining the selection of relevant studies throughout the review process. This iterative nature is characteristic of integrative and narrative reviews, which allow for ongoing adjustments as the analysis develops (18, 21).

While this approach does not follow formal systematic review protocols, it was regarded as appropriate given the heterogeneous and interdisciplinary nature of the topic (public health, engineering, policy, and hospital management). The goal was to identify key themes, strategies, and barriers relevant to German hospitals within the context of international developments, rather than to quantify or meta-analyze specific intervention outcomes.

## 3 Advancing sustainability in healthcare: enhancing well-being, environmental protection, and climate resilience

Healthcare systems have a profound responsibility not only to treat diseases but also to promote human well-being and environmental sustainability. As climate change, pollution, and resource consumption intensify, healthcare institutions must adopt strategies that safeguard both public health and the planet. This section explores the interplay between human well-being and environmental factors, highlighting sustainable construction, energy-efficient solutions, eco-friendly food systems, waste reduction, and climate-conscious healthcare practices. Implementing these measures will contribute to a resilient healthcare system that reduces its ecological footprint while ensuring high-quality patient care.

### 3.1 Human well-being

Physical and mental well-being play crucial roles in overall health. With its primary mission of enhancing, restoring, and preserving the population’s health, the healthcare sector holds a distinctive responsibility for environmental preservation. A healthy environment positively influences well-being, while environmental pollution is implicated in increased morbidity and mortality (22). In 2016, the World Health Organization (WHO) documented that 13.7 million global fatalities resulted from environmental factors (23), with 1.6 million attributed to the direct or indirect effects of chemicals and 4.2 million to air pollution (11). Mitigating environmental pollution (water, air, soil) and mitigating the anticipated global temperature rise are pivotal for disease prevention and contribute to health promotion (Figure 1). Table 1 delineates potential adverse health effects resulting from increased environmental pollution and global temperature rise.

**Table 1:** Potential health impacts of increased environmental pollution and/or global temperature rise.

Global Temperature Rise	Physical and mental stress reactions in the body resulting in increased morbidity and mortality, especially in vulnerable groups. Increase in preterm delivery due to increased resistance in the uterine artery and, therefore, fewer healthy offspring. Spread of infectious diseases typically endemic to warmer regions, e.g., Malaria, Dengue fever. Extreme weather events can lead to physical and psychological changes and damage. Impairment of ecosystems and consequent alteration of food composition. Ozone depletion leads to an increased incidence of skin cancer. Disruption of sleep quality. Destruction of ecosystems. Increase in mental disorders (anxiety, depression). Reduction in work productivity. Reduction in physical work capacity. Reduction in abilities requiring complex cognitive functions. Reduction in motor skills requiring high dexterity.
Air Pollution	Increase in respiratory diseases, e.g., Asthma. Damage to lung function. Disruption of sleep quality. Increased risk of infection. Decrease in physical fitness. Destruction of ecosystems. Increase in mental disorders (anxiety, depression).
Water Pollution and Water Scarcity	Increase in pathogens in drinking water (bacterial/viral) leading to the transmission of infectious diseases. Contamination with harmful substances/(heavy) metals/chemicals can result in poisoning symptoms, autoimmune disorders, and cancer. Destruction of ecosystems. Increase in mental disorders (anxiety, depression).
Soil Pollution	Contamination of food can lead, for example, to gastrointestinal diseases, an increased risk of cancer, or poisoning symptoms. Destruction of ecosystems. Increase in mental disorders (anxiety, depression).

According to (11, 16, 22, 41, 67).

Despite the adverse effects of climate change on human health listed above, healthcare professionals can enhance patient well-being through preventive medical interventions while simultaneously contributing to climate change mitigation by reducing the incidence of diseases. By teaching about a healthy lifestyle that promotes exercise, a balanced diet and the avoidance of harmful habits such as smoking, not only the risk of chronic diseases can be reduced, but also the use of healthcare resources can be minimized by reducing the need for treatment and medication (22, 24, 25). Early diagnosis through regular screening allows diseases to be treated at an early stage, reducing the use of energy and material-intensive therapies (26). Vaccinations for example prevent the spread of infectious diseases, reducing the need for hospitalization and drug therapy. The incidence of HPV-related cervical cancer has already declined worldwide due to the introduction of HPV vaccination and systematic screening for early detection (27). Fewer medical interventions mean lower CO₂ emissions and less medical waste. In the long term, the promotion of human wellbeing through a healthy lifestyle and the prevention of illnesses can help to reduce environmental impact of healthcare and can also make healthcare more sustainable.

### 3.2 Sustainable construction and transportation transition

Sustainable construction and environmentally friendly renovation practices in the hospital sector can enhance energy efficiency, leading to a reduction in resource consumption and greenhouse gas emissions (Figure 1). Approximately 48% of the German population supports the idea of energy-efficient renovations and planning for hospital buildings (28). Energy efficiency enhancements within healthcare facilities can be achieved through various measures (11, 29). The provision of heat or cooling contributes to 25% of a hospital’s greenhouse gas emissions, with approximately 15% attributed to construction and infrastructure (28).

To achieve improved heat efficiency, strategies like the implementation of energy-efficient thermal insulation, adoption of energy-saving technologies including building insulation, heating systems, and ventilation systems with heat recovery, as well as the integration of intelligent building automation systems (e.g., passive cooling through automatic window shading in response to sunlight or temperature rise above a set value; regulation of indoor temperature using electronic sensors on radiators) should be used. Given the absence of standardized guidelines for existing hospitals, best practices should be drawn from the latest advancements in energy efficiency and sustainability. Thus, new hospital buildings should be designed and constructed based on the most up-to-date scientific knowledge to maximize energy performance and environmental sustainability (11, 29, 30).

Furthermore, enhanced spatial planning can optimize area requirements, creating additional green spaces like green roofs to reduce the carbon footprint (Figure 1). This reduction in greenhouse gas emissions contributes to a decreased ecological footprint for hospitals, and adherence to high construction standards can prolong the lifespan of buildings.

The transportation of medical products, food, and the transportation of people (patients and healthcare workers) significantly contributes to greenhouse gas emissions in the healthcare sector. Consequently, companies, including those in the hospital sector, are legally mandated to identify and address environmental risks in both upstream and downstream value chains (15).

To mitigate the environmental impact, it is recommended to prioritize the use of sustainable energy sources in all healthcare transportation activities, such as patient transport and the distribution of medications or other medical products (11). Hospitals can further encourage sustainable transportation options for staff and patients by providing facilities like bicycle racks and electric vehicle charging stations (11, 30). Initiatives promoting carpooling and using public transportation, possibly through financial incentives, can also contribute to reducing the carbon footprint (Figure 1) (31).

The German Hospital Association emphasizes the urgent implementation of digitalization and telemedicine as a priority goal for the current legislative period of the German House of Representatives (“Bundestag”) (32). The expansion of digital structures within the hospital sector not only enhances patient care and safety but also aligns with sustainable practices. Adopting paperless documentation and reporting helps minimize paper consumption (29, 31). Additionally, eliminating unnecessary hospital visits for appointments that do not require physical presence can further contribute to a reduction in CO2 emissions.

### 3.3 Energy transition and climate protection

The findings of a recent representative survey indicate that approximately 50% of the German population expresses a desire for reduced energy consumption within the German hospital system (28). Given the healthcare sector’s dependence on a stable and reliable power supply to ensure continuous patient care (11, 31), the imperative to enhance energy efficiency has become pivotal, particularly with the demographic shift leading to a rise in age-related illnesses, intricate treatment procedures, diagnostic technologies, and escalating energy costs (Figure 1).

Hospitals can strategically adopt renewable energy sources, such as using energy from wind and hydropower, and actively promote the installation of solar panels to utilize solar energy for their operational needs (Figure 1) (29). This shift towards renewable energy sources in the healthcare sector aligns with the expectations of 43% of the German population (28). Improving electricity consumption efficiency involves the implementation of time-optimized LED lighting, the utilization of energy-efficient information technology and electrical appliances, and the optimization of the use of large devices, such as power-intensive equipment, in radiology departments (29).

### 3.4 Sustainable agricultural and food systems

The hospital sector in Germany, with its approximately 483,000 beds in the year 2021 (33), assumes a pivotal role in the alimentation system. Food systems are integral to achieving sustainability goals, encompassing the entirety of the food supply chain, including production, distribution, preparation, and consumption, while considering additional influential factors such as the environment, resources, and infrastructure (11, 30, 34). Food production within the hospital sector involves resource consumption and has the potential to generate emissions and waste throughout each phase of the supply chain (11, 34).

In the context of sustainability, the creation of regional partnerships or network structures is essential for promoting sustainable food supply and usage for both patients and staff (Figure 1). Practices such as minimizing transportation distances and incorporating fresh seasonal products from organic farms not only contribute to overall health and support the local economy but also diminish the reliance on imported foods, consequently reducing the CO_2_ footprint (29). Strategies like reducing portion sizes not only curtail food waste but, when combined with a decrease in meat consumption, can further mitigate the hospitals CO_2_ footprint (11, 31). Simultaneously, educational initiatives and informative programs directed at patients and staff can facilitate the introduction and expansion of sustainable food procurement and utilization practices in private households. This, in turn, leads to a sustained and more significant reduction in the CO_2_ footprint, contributing substantively to environmental conservation.

Upon deeper consideration, a nutritionally balanced, predominantly plant-based diet not only plays a pivotal role in disease prevention but also possesses therapeutic potential (30). On a global scale, the prevalence of obesity is rising, contributing significantly to the increasing burden of obesity-related comorbidities. The management and treatment of these conditions necessitate substantial healthcare resources, imposing considerable economic and societal strain. Consequently, numerous organizations have established guidelines advocating for a climate-friendly diet (24, 30). Integrating these recommendations into patient consultations and implementing them in hospital settings for both patient care and staff nutrition could contribute to improved health outcomes while simultaneously promoting environmental sustainability (35).

### 3.5 Pollutants-free environment

To effectively safeguard the environment and public health from the adverse effects of pollutants, it is imperative to implement and continually enhance an efficient and sustainable waste management system. The concept of a sustainable circular economy aims to minimize waste or optimize waste management practices. The hospital sector, generating 7 to 8 tons of waste per day, stands as one of the largest waste producers in Germany (36). Approximately 15% of the waste generated by the healthcare sector has the potential to pose hazards to both humans and the environment, being infectious, toxic, or radioactive in nature (11). Consequently, the hospital sector holds significant potential to reduce various types of waste (Figure 1). Adequate waste separation facilitates the reintegration of valuable resources into the economic cycle, contributing to resource conservation.

There are five main strategies for waste reduction: reduction, reuse, recycling, rethinking, and research (37). The active involvement of on-site staff is crucial, as they should regularly contribute their ideas and proposed strategies for waste reduction in their respective institution (31, 37). In general, prioritizing the substitution of hazardous substances with environmentally friendly alternatives and ensuring the proper recycling and reutilization of non-hazardous substances is essential (11).

The hospital sector’s main contributors to waste are operating rooms and hemodialysis wards (37). Approximately 15% of a hospital’s greenhouse gas emissions are associated with single-use or reusable medical products (28). In the healthcare sector, 30–70% of waste is generated in the interventional sectors, with the endoscopic and anaesthesiology departments being the primary contributors (37). An example is the protective clothing for medical personnel, which contains polypropylene (PP), with a single FFP-2 mask containing 11 g of PP (37). Although PP itself is not toxic, its production results in high CO_2_ emissions, and being non-biodegradable, a significant amount of PP contributes to microplastic pollution in drinking water and oceans (38). Transitioning to 100% biodegradable products while ensuring safety for patients and medical personnel is a desirable goal.

Hospitals can reduce the use of disposable products, opt for bulk packaging over individual ones, adopt rechargeable batteries instead of disposable ones, and introduce reusable medical devices or materials (31). Hospitals are required to follow the waste hierarchy established in the Circular Economy Act: 1. Waste prevention; 2. Energy or material recovery; 3. Appropriate disposal methods (e.g., incineration, landfill sites) (39).

Efficient recycling initiatives and take-back programs for non-medical materials, including plastics, glass, paper, and electrical appliances, and investments in waste processing facilities can effectively reduce generated waste and reintegrate vital raw materials into the economic cycle (31, 39).

An additional noteworthy and promising approach is the concept of “Medical Remanufacturing,” which involves the reprocessing of single-use medical products like electrophysiology catheters. This process ensures the preservation of both functionality and restoration of sterility, thereby ensuring patient safety (31). Legally regulated under Article 17 of the Medical Device Regulation MDR (EU) 2017/745, the Medical Remanufacturing process mimics all phases of new production for each product but with significantly lower resource consumption and reduced greenhouse gas emissions (40). Germany, alongside the United States, stands out as a pioneer in this innovative field.

## 4 Implementation of sustainability in the healthcare sector

Given the significant greenhouse gas emissions associated with the healthcare sector, a comprehensive analysis of emission sources and the identification of effective mitigation strategies are imperative to reducing its environmental impact.

Besides effective waste management, several measures can contribute to preventing environmental pollution, including the preservation of clean air, water, and soil pollution (11, 41). The adoption of innovative technologies, such as air filters, can effectively capture pollutant particles, and the implementation of air quality monitoring systems can offer early detection of potential air pollution events (41).

Fundamentally, efforts should be made to reduce or eliminate the use and release of environmentally harmful chemicals. A comprehensive catalogue of potentially toxic chemicals or processes for each specific area should be compiled, and a shift towards environmentally friendly alternatives is imperative. Particularly within the healthcare sector, adopting environmentally friendly, low-pollution, and biodegradable disinfectants can mitigate the release of potentially harmful chemicals into the environment (29). Another strategy to minimize chemical usage is the utilization of saturated industrial dry steam for cleaning floors and surfaces (29). In radiology departments, transitioning from analogue to digital radiography is recommended as it helps avoid the use of environmentally harmful chemicals (29, 31).

The anaesthesiology departments, particularly, play a significant role in contributing to greenhouse gas emissions due to the utilization of volatile anesthetics. This contributes to the further depletion of the ozone layer and the acceleration of global temperature rise. Desflurane, for instance, surpasses the greenhouse effect of CO2 by 2,540 times, while a similarly effective anesthetic like sevoflurane only exceeds the greenhouse effect of CO2 by 130 times (42). Consequently, a straightforward transition from desflurane to sevoflurane, without requiring additional investment measures, can lead to a substantial reduction in greenhouse gas emissions from hospitals (42, 43). Implementing low-flow anesthesia to decrease anesthetic volume and utilizing efficient anesthesia gas filters for the absorption of climate-damaging anesthetics can further contribute to greenhouse gas reduction (31, 43).

Maintaining a clean water supply is essential for upholding, fostering, or restoring the health and well-being of the population (Figure 1) (11). To minimize water pollution, it is crucial to appropriately segregate and dispose of toxic or hazardous waste, such as carcinogenic or radioactive substances. The use of environmentally friendly cleaning and disinfecting agents aids in preserving water quality and preventing soil pollution. The implementation of water purification systems can effectively filter pollutants from wastewater, thereby maintaining the quality of groundwater (11).

When implementing any of the sustainability measures listed above within the healthcare sector, certain factors act as catalysts for implementing appropriate processes, while others act as barriers. Table 2 describes both promoting elements and obstacles in implementing sustainability measures.

**Table 2:** Potential facilitating factors and barriers in the implementation of sustainability measures.

Facilitating Factors	Barriers
Active involvement of employees (idea generation, implementation, dissemination of strategic measures) Training/education Generating awareness Sustainability certifications as quality indicators Identifying sustainability measures as long-term profit-enhancing Sustainability measures as an image-enhancing factor	Knowledge gaps Unwillingness Lack of leadership at the executive level Staff shortage: Insufficient time/staff for implementation Inadequate funding by the German decentralized federal system Technical complexity of innovative sustainable projects Infeasibility of a uniform concept for sustainability implementation due to different structural and disciplinary orientations of individual hospitals

According to (11, 29, 31, 37, 68).

Environmental pollution exerts a detrimental influence on individuals’ productivity, diminishes the Gross Domestic Product (GDP), and translates into a 6.2% loss of global economic output (41). Mitigating environmental pollution can result in substantial net gains for both the economy and human health (41). Calculations show that, in the United States, a $1 investment in sustainability measures of air pollution control in the healthcare system led to long-term benefits of 30$ (95% CI $4 to $88), which accounts for an aggregated benefit of 1.5 trillion after an investment of $65 billion (41).

These factors emphasize the significance of active employee involvement, continuous education and training, awareness generation, and recognizing sustainability measures as drivers of long-term profit and a positive public image. Conversely, challenges such as knowledge gaps, unwillingness, lack of leadership, staff shortage, insufficient funding, technical complexity, and the diversity of hospital structures and disciplines can impede the effective implementation of sustainability measures in the healthcare sector.

The prompt implementation of effective sustainability measures in hospitals necessitates a commitment from the leadership level, complemented by the engagement of dedicated and interested employees across all professional groups and organizational tiers (Figure 1). The appointment of a „Sustainability Manager “and regular training for the entire staff on climate protection can play a pivotal role in facilitating this process (31).

A notable impediment in the execution and enforcement of sustainability measures in national and international healthcare systems is the scarcity of qualified and committed personnel. Currently, healthcare systems globally are struggling with a shortage of personnel and excessive workloads, a situation anticipated to exacerbate in the future. Recent data from Germany in 2022 underscores this challenge: 72% of doctors and nurses report physical strain due to their work, and the German healthcare system has 290,000 open positions (44). This trend is expected to deteriorate in the coming years. Germany is projected to face a shortage of skilled workers in healthcare, reaching 1.8 million by 2035 (44), and the United Nations foresees a global shortage of 18 million healthcare sector workers by 2030 (45). The personnel shortage will impede overloaded employees from finding the necessary time to address sustainability issues at their level and within their field of activity.

The automation of processes and the digitalization of the healthcare sector offer a dual solution in addressing sustainability challenges. Firstly, these measures directly contribute to achieving sustainability goals, such as reducing paper consumption. Secondly, they alleviate the workload on healthcare professionals, enabling them to dedicate their working hours to sustainability initiatives.

Implementing sustainability activities can enhance the image of healthcare professions, contributing to the mitigation of the shortage of skilled workers. Moreover, hospitals demonstrating excellence in sustainability can leverage a positive image to attract and retain qualified professionals.

The implementation of sustainability reporting, as mandated by an EU directive and aimed for integration into national law by 2024 (28, 46), is a critical undertaking in the healthcare sector. It necessitates timely attention and the development of a comprehensive sustainability strategy. Establishing concrete sustainability goals across short-, medium-, and long-term timelines, coupled with the prioritization of recommendations, is essential and should undergo regular reviews. The findings should be consolidated in an annual sustainability report by each healthcare institution.

The initial phase involves conducting a “Status Quo” assessment to evaluate the existing sustainability status of each hospital, facilitated by a dedicated and qualified team (Figure 1) (11). Early involvement of non-medical key sectors, including the food industry and supply chain partners, is crucial to ensure a holistic approach (11).

Environmental and sustainability certifications can function as additional quality indicators within healthcare. Ideally, these certifications should be linked to financial benefits, such as a specific DRG code usable by recognized clinics or departments. This financial incentive aims to encourage hospitals to participate in sustainability programs actively (Figure 1). Justifying the financing through health insurance contributions from the insured population is grounded in the understanding that environmental protection plays a pivotal role in disease prevention, aligning with preventive measures integrated into the service catalog of health insurance funds.

Within the healthcare sector, embracing sustainable practices not only enhances the image of hospitals committed to environmental responsibility but also positions them favorably in securing financing and obtaining credit approvals for ongoing investments from financial institutions (28).

## 5 Financing and economic viability of sustainability measures

The comprehensive adoption of sustainability measures contributes significantly to improving the cost-efficiency of hospitals, enhancing financial stability, equitably distributing resources, and ensuring a high quality of life for future generations. The healthcare sector can assume a leadership role in society by instituting and implementing structures and processes that advance sustainability.

The implementation of an energy management system conforming to the internationally recognized ISO 50001 standard facilitates the augmentation of energy efficiency by identifying energy consumption patterns and potential areas for savings (31). Although this may involve initial higher investments for hospitals or the healthcare sector, it not only stimulates economic activity but also, in the long term, leads to a reduction in energy consumption and operational costs, resulting in cost savings for the healthcare system. According to estimates from the Viamedica Foundation, a large hospital with an annual budget of 500 million Euros could save approximately 30% of energy and water costs, translating to around 3 million Euros per year (12). The financial resources thus freed up over the long term can be directed towards implementing innovative technologies that further promote sustainability, improving working conditions and job satisfaction for the healthcare staff, and improving patient care.

The majority of the sustainability improvement measures are not fully integrated into the German hospital system. Despite their long-term cost-effectiveness, these measures initially necessitate significant financial investments. In Germany, the individual federal states are responsible for financing hospital investments. The annual benchmark, calculated by the Institute for the Hospital Remuneration System (InEK) to determine investment needs, stands at approximately 7 billion Euros (32). This figure should be expanded to encompass funds for digitalization and the investment requirements of university hospitals. Nevertheless, the federal states contribute less than half of this amount (32). Consequently, there are substantial delays in implementing essential sustainability measures within the hospital sector, posing the risk of irreversible consequences for both human health and ecosystems. In this context, the federal government could establish an incentive system that rewards states that provide investment funds that meet the actual investment requirements (Figure 1).

## 6 Comparative insights into sustainability in healthcare systems

Sustainability in healthcare is an evolving field, with hospitals worldwide adopting diverse strategies to enhance energy efficiency, reduce waste, integrate sustainable food systems, and implement green hospital designs. However, regulatory environments, financial structures, and policy frameworks significantly shape how sustainability measures are adopted. This study examines the sustainability challenges and solutions in German hospitals and compares them with findings from similar studies in the UK, USA, Netherlands, Canada, Australia, Ireland, and Middle Eastern and European healthcare systems.

By drawing insights from empirical studies and real-world case analyses, this section highlights key similarities, differences, and lessons learned that could inform policy recommendations and future research for Germany’s hospital sustainability agenda.

### 6.1 Energy efficiency and climate protection in hospitals

Energy efficiency is a critical component of hospital sustainability, with this study identifying renewable energy adoption (solar, wind), energy-efficient hospital designs, and LED lighting as key strategies for German hospitals. However, financial constraints and regulatory barriers make large-scale energy retrofits difficult.

Comparatively, Piubello Orsini et al. found that green energy procurement significantly improved NHS hospital sustainability, emphasizing the importance of national policies in accelerating change (Table 3) (47). Chen-Xu et al. conducted a meta-analysis of global hospital efficiency interventions, finding that LED lighting and HVAC optimization reduced energy use by 36% (Table 3) (48). This aligns with findings in this study but highlights that German hospitals face higher regulatory complexity and funding shortages, unlike UK and other international healthcare systems that benefit from structured green incentives. Additionally, Alotaiby and Krenyácz studied energy efficiency in Middle Eastern and European hospitals, showing that hospitals integrating refrigerant flow technology and renewable-diesel hybrid systems achieved significant energy savings (49). While German hospitals could theoretically adopt similar approaches, decentralized hospital governance and varied regional funding models present implementation barriers.

**Table 3:** Comparative analysis of sustainability measures in healthcare.

Study	Country	Focus area	Key findings	Barriers identified	Unique contributions
Current study	Germany	Comprehensive sustainability measures in hospitals	Identifies key sustainability objectives and implementation barriers	Workforce shortages, decentralized funding, regulatory complexity	Policy recommendations tailored for German hospitals
Piubello Orsini et al. (47)	UK	Climate change mitigation in hospitals	Green energy procurement improved NHS hospital sustainability	High initial investment	Practice-based view for green hospital management
Frassanito et al. (69)	Italy	IoT and AI for hospital energy efficiency	AI-driven HVAC system upgrades reduced energy consumption	Initial setup complexity	Case study on AI-based hospital energy management
Chen-Xu et al. (48)	International	Energy efficiency in hospitals	LED lighting and HVAC optimization reduced energy use by 36%	Implementation costs	Meta-analysis of global hospital efficiency interventions
Alotaiby and Krenyácz (49)	Middle East and Europe	Energy efficiency in healthcare institutions	Variants of refrigerant flow tech and renewable-diesel hybrid systems improved hospital energy savings	Regional energy policies, high upfront costs	Identifies cross-country hospital energy solutions
Lee et al. (52)	USA	Waste management in hospitals	Waste segregation and recycling reduced medical waste by 30%	Upfront costs, lack of incentives, and the complexity of shifting towards net-zero carbon goals	Framework for decarbonizing healthcare by integrating climate change mitigation strategies
Wierda et al. (56)	Netherlands	Sustainable food systems in hospitals	Characterized food environments in hospitals and long-term care facilities	Lack of national guidelines for food sustainability, staff resistance and logistical challenges	Comparative analysis of hospital vs. long-term care food sustainability
Timmermann and Wild (70)	Germany	Sustainability transitions in university hospitals	Emphasizes carbon literacy and green healthcare education	Lack of institutional incentives	Ethical approach to hospital sustainability
Delgado et al. (71)	USA	Integrating health and energy efficiency	Framework to evaluate health and energy efficiency in hospitals	Complexity in implementation	Healthy Buildings Toolkit for hospital energy strategies
Singh et al. (65)	USA	Decarbonization in hospitals	Hospitals account for 8.5% of US greenhouse gas emissions	Financial and operational barriers	Call to action for hospital emissions reporting
Angelov et al. (58)	Ireland	Green hospital transportation	Only 15% of hospitals had EV charging, and 36% had bike parking	Limited infrastructure funding	Created a novel sustainable hospital transport ranking system
Gurieff et al. (59)	Australia	Hospitals as sustainable energy hubs	Proposed hydrogen-based hospital energy system to reduce emissions	High initial investment	Conceptualized hospitals as net-zero energy hubs
Spoyalo et al. (57)	Canada	Sustainable food choices in hospital cafeterias	Behavioral nudges increased plant-based food purchases	Consumer resistance to change	Studied availability and salience effects on hospital food sustainability
Parekh (66)	USA	LEED Certification in hospitals	Healthcare projects struggle with Energy and Atmosphere (EA) criteria in LEED ratings	High energy demand of hospitals	Comprehensive analysis of LEED-HC v4.1 adoption in US hospitals

Unlike the UK hospitals, which benefit from structured national funding mechanisms for energy transitions (50), Germany, while making significant strides in renewable energy and energy efficiency through initiatives like the €500 billion infrastructure fund and the International Climate Initiative, still lacks a cohesive, hospital-specific financial support system (51). This absence of centralized funding for hospital energy transitions may contribute to slower adoption compared to other countries. Developing national-level green energy procurement strategies tailored to healthcare facilities could accelerate sustainability efforts in German hospitals.

### 6.2 Hospital waste management and circular economy

This study highlights that German hospitals generate 7–8 tons of waste per day, with 15% being hazardous, yet they lack centralized policies to standardize waste reduction efforts. Lee et al. reported that waste segregation and recycling reduced medical waste by 30% in American hospitals but encountered staff resistance and high disposal costs (Table 3) (52). Additionally, many US hospitals have successfully integrated circular economy principles due to federal waste management incentives (Table 3) (52).

Germany has a well-established waste management framework under the Waste Management Act (KrWG) (53), which promotes recycling and waste reduction, but it lacks a centralized, hospital-specific policy for circular economy integration. In contrast, countries like the UK have a structured NHS Waste Reduction Strategy that encourages waste segregation and sustainability in healthcare, supported by financial incentives and regulatory guidelines (54). Similarly, Japan has a robust Extended Producer Responsibility (EPR) system that holds producers accountable for the end-of-life management of products, including medical equipment, contributing to sustainable waste management in hospitals (55). While Germany has strong national policies on waste management, the healthcare sector would benefit from a tailored framework to enhance waste reduction and circular economy practices in hospitals, similar to those in the UK and Japan.

### 6.3 Sustainable food systems in hospitals

This study emphasizes reducing hospital food emissions through local sourcing and plant-based diets but notes fragmented sustainability efforts due to high procurement costs and lack of incentives.

Wierda et al. explored barriers to sustainable food adoption in Dutch hospitals, revealing that stakeholder engagement and policy-driven food procurement programs improved food sustainability (Table 3) (56). This supports the findings in this study that stronger governmental backing is needed in Germany to facilitate sustainable hospital food systems.

Similarly, Spoyalo et al. tested behavioral interventions (salience and availability) in hospital cafeterias, finding that making plant-based meals more visible increased their selection by staff and patients (Table 3) (57). This aligns with this study’s suggestion that behavioral nudges could complement policy-driven changes to encourage sustainable food adoption in German hospitals.

Sustainability-driven food procurement strategies and behavioral interventions could help increase the adoption of low-carbon meal options in German hospitals. However, cost barriers remain a significant challenge, requiring government-backed hospital food sustainability guidelines.

### 6.4 Green hospital design and sustainable transportation

The German hospital sector is gradually incorporating sustainable infrastructure. However, it lags behind countries like Australia and the UK due to financial constraints and regulatory barriers.

Angelov et al. reported that only 15% of hospitals were equipped with EV charging, and 36% had bike parking, indicating that sustainable transportation infrastructure remains underdeveloped even within advanced healthcare systems (Table 3) (58). This study mirrors these findings as German hospitals struggle with funding for EV charging stations and sustainable transport initiatives. However, the UK and Australian hospitals have taken more structured steps, such as integrating low-emission ambulance fleets and providing financial incentives for staff cycling initiatives. Gurieff et al. conceptualized hospitals as sustainable energy hubs, demonstrating that hydrogen-based hospital energy systems can significantly reduce emissions (Table 3) (59).

While Australia has begun experimenting with hydrogen-based hospital energy solutions, German hospitals continue to rely predominantly on conventional grid-based energy sources. Encouraging green hospital transport policies, such as incentivizing EV charging stations and bike parking facilities, may facilitate bridging this disparity.

### 6.5 Decarbonization and climate-friendly hospitals

This study discusses the role of hospitals in reducing their carbon footprint, highlighting that German hospitals encounter financial limitations and policy gaps that hinder the adoption of sustainability practices. Countries like Australia, the United States, and the United Kingdom have developed structured, hospital-specific carbon neutrality goals. For example, Australia’s Sustainable Health Strategy 2020–2030 targets net-zero emissions for hospitals by 2050 (60). In the United States, the Cleveland Clinic (61) and University of California Health System have set clear carbon neutrality goals for 2050 and 2025 (62), respectively, supported by initiatives from Health Care Without Harm (63). Additionally, the NHS in the UK has committed to net-zero by 2040 through its NHS Climate Change Strategy, focusing on energy efficiency and sustainable procurement (64).

Singh et al. found that hospitals contribute 8.5% of total US greenhouse gas emissions and proposed system-wide decarbonization strategies (Table 3) (65). Unlike the US model, where centralized climate policies guide emission reductions, German hospitals lack a standardized carbon reduction framework, making it challenging to enforce uniform sustainability goals across different states.

Furthermore, Gurieff et al. conceptualized hospitals as sustainable energy hubs, proposing hydrogen-based hospital energy systems to cut emissions (Table 3) (59). This contrasts with German hospitals, which are still in the early stages of decarbonization efforts. Integrating renewable energy with hospital energy networks, as seen in Australia’s model, could serve as a blueprint for future sustainability strategies in Germany.

Germany lacks a structured decarbonization roadmap for hospitals. Developing hospital-specific carbon neutrality goals, like those seen in Australia, the UK, and the US, could be a critical step in accelerating Germany’s healthcare sustainability transition.

### 6.6 LEED certification and hospital sustainability metrics

This study highlights that hospital sustainability measures in Germany lack standardization, making it difficult to compare performance across facilities.

Parekh et al. analyzed LEED-HC v4.1 certification scores across 120 hospitals, showing that many hospitals struggled with meeting the Energy and Atmosphere criteria (Table 3) (66). This aligns with this study’s findings that German hospitals struggle to meet energy efficiency benchmarks. However, the US model provides a structured certification framework, whereas Germany lacks a national hospital sustainability rating system. Developing a LEED-equivalent certification in Germany could help benchmark hospital sustainability performance and drive improvements.

This study highlights the absence of a standardized sustainability rating system for German hospitals, making it difficult to track progress and benchmark performance. Introducing a national hospital sustainability certification framework, similar to LEED-HC (USA), could help standardize and monitor sustainability progress in German hospitals.

## 7 Practical implications of this study

Sustainability in healthcare is an increasingly critical topic, yet research in this domain faces several persistent challenges. One of the most pressing issues is the lack of empirical data on the effectiveness of various sustainability interventions in hospitals. While many initiatives, such as renewable energy adoption, waste reduction programs, and sustainable food systems, have been implemented in different countries, there is still limited research on their long-term impact and cost-effectiveness. Without robust data, it becomes difficult to develop evidence-based policies and best practices for hospital sustainability.

Another significant barrier is the absence of standardized sustainability benchmarks across healthcare systems. Unlike sectors such as energy or transportation, where sustainability metrics are more clearly defined, hospitals operate within highly complex environments with diverse regulatory and operational constraints. In Germany, the decentralized healthcare funding model further complicates the implementation of sustainability measures, as hospitals must navigate varying regional policies and financial structures. This fragmentation makes it challenging to compare sustainability performance across hospitals and track progress on a national scale.

Financial and logistical constraints also pose substantial challenges. Many hospitals struggle to secure funding for sustainability initiatives, particularly for large-scale infrastructure upgrades such as energy-efficient building designs, solar installations, and circular waste management systems. Additionally, even when funding is available, the implementation of these solutions can be logistically complex, requiring specialized expertise and long-term commitment from hospital management.

Beyond financial and logistical hurdles, behavioral and cultural resistance among hospital staff can significantly impact sustainability adoption. Many hospital employees, from administrators to medical personnel, may view sustainability initiatives as additional burdens rather than essential priorities. Without comprehensive staff engagement and training programs, there is a risk that sustainability measures will not be fully integrated into daily hospital operations.

To address these challenges, future research should focus on longitudinal studies that assess the long-term environmental and economic impact of sustainability initiatives in hospitals. By gathering data over extended periods, researchers can provide stronger evidence for the benefits of sustainability interventions, making it easier for hospitals to justify their adoption. Additionally, there is a need to develop standardized sustainability metrics that allow for cross-country comparisons of hospital sustainability performance. Establishing such benchmarks will help policymakers and healthcare administrators track progress, identify best practices, and set realistic sustainability targets.

Another key area for future research is the cost-effectiveness of circular economy models in healthcare waste management. While some hospitals have successfully implemented recycling and waste reduction programs, the broader economic benefits of these initiatives remain unclear. Understanding the long-term savings associated with circular economy approaches can encourage more hospitals to adopt waste reduction strategies. Additionally, future studies should explore staff training programs and behavioral interventions to foster a culture of sustainability within hospitals. Effective training programs can help overcome staff resistance, ensuring that sustainability becomes an integral part of hospital operations.

To accelerate the transition toward sustainable hospitals, policymakers and healthcare administrators must implement concrete policy changes. One of the most impactful steps would be to establish sustainability mandates for hospitals, similar to those implemented in the UK’s National Health Service (NHS), where hospitals are required to meet climate targets and report on their sustainability progress. Financial incentives such as tax benefits, grants, and low-interest loans should also be introduced to support hospitals in adopting renewable energy, waste reduction programs, and green infrastructure projects.

Sustainable procurement policies should also be prioritized, particularly in hospital food sourcing. By promoting local and organic food procurement, hospitals can reduce their environmental footprint while ensuring healthier meals for patients and staff as co-benefits. Additionally, investing in digital healthcare solutions such as telemedicine and electronic documentation systems can significantly reduce hospital carbon footprints by minimizing the need for travel and paper-based processes, further contributing to overall sustainability goals.

By integrating these research directions and policy recommendations, German hospitals can transition toward more sustainable, cost-effective, and environmentally responsible healthcare systems. A structured approach that combines empirical research, financial incentives, standardized metrics, and staff engagement will be essential to making hospital sustainability a reality.

## 8 Strengths and limitations

One strength of this work is the integrative and narrative review approach, which allowed for an in-depth exploration of sustainability practices in German hospitals and beyond by synthesizing a wide range of literature across different disciplines. This flexibility is particularly beneficial when addressing complex and interdisciplinary topics, where systematic reviews might be too rigid (18). Additionally, integrative reviews excel at identifying emerging trends and knowledge gaps, making them especially useful for new or under-researched topics like sustainability in healthcare. This review highlights the evolving landscape of sustainability practices in hospitals and offers a foundation for future research directions (21).

Nevertheless, some limitations must be acknowledged. Due to the non-systematic nature of the review, there is a potential for selection bias. While efforts were made to include a broad range of studies, the process was not as exhaustive or reproducible as in systematic reviews. The inclusion of studies was based on thematic relevance, which may have led to the exclusion of some potentially valuable studies (18, 20). Integrative reviews do not adhere to strict inclusion and exclusion criteria, making them harder to replicate. This can limit the reproducibility of the findings and introduce subjectivity into the selection process (20). Unlike systematic reviews, which often use meta-analysis to synthesize quantitative data, this narrative review is qualitative and does not attempt to quantify the effects of sustainability interventions. While this is appropriate for the exploratory nature of the review, it means that the conclusions drawn are more subjective and context-dependent (18).

## 9 Conclusion

Hospitals play a pivotal role in sustainability transitions due to their significant energy consumption, waste production, and impact on public health. However, the integration of sustainability measures in German hospitals remains slow, hindered by regulatory fragmentation, financial constraints, and the lack of a standardized sustainability framework. Unlike the UK, USA, and Australia, where structured incentives and policies have facilitated progress, Germany’s fragmented approach makes it challenging to benchmark sustainability efforts and implement large-scale changes effectively.

This study identifies key barriers and opportunities for enhancing sustainability in German hospitals, particularly in energy efficiency, waste management, sustainable food procurement, and green infrastructure. The findings highlight the financial and structural hurdles that hinder the adoption of renewable energy solutions, in contrast to the centralized funding and policy-driven models seen in the NHS. Waste management remains fragmented due to a lack of national policy coherence, whereas US hospitals have successfully implemented circular economy approaches to reduce medical waste. Sustainable food systems offer another area for improvement, with Dutch and Canadian hospitals demonstrating the effectiveness of policy-driven procurement and behavioral interventions in promoting plant-based and locally sourced meals. Furthermore, Germany lags behind Australia and Ireland in green hospital design and sustainable transportation, limiting its ability to reduce emissions and improve environmental efficiency.

This research provides valuable insights for policymakers, hospital administrators, and sustainability experts by identifying key sustainability gaps in German hospitals and offering evidence-based recommendations on hospital decarbonization, circular economy integration, and food sustainability. This study offers a strategic roadmap for improving Germany’s hospital sustainability practices by analyzing successful sustainability models from international healthcare systems.

To accelerate progress, future research and policy efforts should focus on developing a national hospital sustainability framework, similar to LEED-HC (USA), to standardize and benchmark performance. Financial incentives and funding models, such as green hospital investment funds and public-private partnerships, should be created to ease the transition to renewable energy and waste reduction programs. Expanding behavioral interventions and procurement policies for sustainable hospital food systems can ensure that hospitals prioritize healthy, low-carbon meal options. Encouraging pilot projects that integrate energy efficiency, carbon reduction, and circular economy solutions will foster collaborations between hospitals, policymakers, and researchers, driving innovation and long-term sustainability.

By adopting these strategies, Germany can significantly enhance hospital sustainability, reduce healthcare-related emissions, and position itself as a leader in sustainable healthcare practices. Future research should evaluate the long-term economic and environmental impact of these interventions, ensuring that hospitals not only deliver high-quality healthcare but also contribute to a greener, more sustainable future.
